# The relationship between anthropometric indices and non-alcoholic fatty liver disease in adults: a cross-sectional study

**DOI:** 10.3389/fnut.2024.1494497

**Published:** 2025-01-07

**Authors:** Mina Radmehr, Reza Homayounfar, Abolghasem Djazayery

**Affiliations:** ^1^Department of Nutrition, Science and Research Branch, Islamic Azad University, Tehran, Iran; ^2^National Nutrition and Food Technology Research Institute, Shahid Beheshti University of Medical Sciences, Tehran, Iran; ^3^Noncommunicable Diseases Research Center, Fasa University of MedicalSciences, Fasa, Iran; ^4^Department of Community Nutrition, School of Nutritional Sciences and Dietetics, Tehran University of Medical Sciences, Tehran, Iran

**Keywords:** non-alcoholic fatty liver, anthropometric indices, a body shape index, body roundness index, NAFLD

## Abstract

**Background:**

Non-alcoholic fatty liver disease (NAFLD) is a widespread liver condition associated with diabetes, metabolic syndrome, and cardiovascular diseases, yet public awareness remains low. Early detection of risk factors is crucial, but liver biopsy, the diagnostic gold standard, is invasive and costly. Non-invasive anthropometric indices provide a safer alternative. This study examines these indices to identify the most reliable predictor of NAFLD in adults.

**Methods:**

In the present cross-sectional study, we used the Fasa Cohort Data, conducted on about 10,000 people, of whom 1,047 were diagnosed with NAFLD. NAFLD diagnosis in this study was confirmed by physicians based on medical history and ultrasonographic evaluations, ensuring accurate and reliable identification of cases. General, anthropometric, and dietary assessments were performed using interviews, tools, and valid questionnaires. Biochemical evaluation was also done. Waist-to-hip ratio (WHR), waist-to-height ratio (WHtR), Body mass index (BMI), a body shape index (ABSI), body roundness index (BRI), and visceral fat index (VAI) were also calculated using these measurements and formulas. This study used descriptive tests, binary logistic regression, and ROC curve analysis.

**Results:**

In both crude and adjusted models, significant associations were found between WHR, WHtR, BMI, and VAI with NAFLD. ROC analysis revealed that WHtR and BMI were the most accurate predictors of NAFLD in both genders (WHtR: men AUC = 0.750, women AUC = 0.702; BMI: men AUC = 0.754, women AUC = 0.701). BRI showed significant accuracy, but WHR (men: AUC = 0.727, women: AUC = 0.640) and VAI (men: AUC = 0.621, women: AUC = 0.622) were less effective. ABSI demonstrated poor predictive power (men: AUC = 0.530, women: AUC = 0.505) and is not recommended for NAFLD prediction.

**Conclusion:**

Based on the findings, BMI and WHtR emerge as the most practical and accessible indicators for early screening of NAFLD in both men and women, while ABSI shows minor effectiveness in identifying the disease.

## 1 Introduction

Non-alcoholic fatty liver disease (NAFLD) is a clinical pathological condition characterized by fat deposition in liver parenchymal cells. The pathological picture is similar to alcohol-induced liver damage but occurs in people without a history of chronic alcohol consumption (1). NAFLD has become an emerging public health issue with high prevalence worldwide, and its incidence is expected to increase rapidly in the future, affecting one-third of the population (2, 3). NAFLD is a significant health concern and the most common form of liver disease worldwide (4). It is a condition defined by a considerable accumulation of fat (5–10%), especially triglycerides (TGs) in liver cells, in the absence of significant chronic alcohol consumption less than 20 grams per day in men and 10 grams per day in women (5–7). This disease ranges from simple steatosis to non-alcoholic steatohepatitis and sometimes cirrhosis and hepatocellular carcinoma (8). In addition to increasing rates of obesity, based on epidemiological data, approximately one-quarter of patients with NAFLD progress to steatohepatitis with fibrosis, which can lead to critical liver-related complications and death (9, 10).

On the other hand, NAFLD is closely associated with high rates of several disorders, including diabetes, metabolic syndrome (MetS), and cardiovascular diseases (CVDs) (2, 11). Similar to the characteristics of other chronic diseases, NAFLD usually has a long course of disease, which has a low level of public awareness despite the many complications (12–15). Additionally, visceral fat (fat surrounding internal organs) and subcutaneous fat have been closely linked to liver health and play specific roles in NAFLD development. Recent research has shown that visceral and subcutaneous fat reductions correlate with a decrease in liver fat (correlation coefficients ranging from 0.33 to 0.49), underscoring the importance of these specific fat types in NAFLD prediction (16). Moreover, visceral fat (fat surrounding internal organs) and subcutaneous fat play specific roles in NAFLD development and progression (17, 18). Another study noted that visceral fat accumulation independently correlates with liver inflammation and advanced fibrosis, underscoring its impact on NAFLD progression even when controlling for factors like insulin resistance (19). Because the damage caused by NAFLD is often chronic and asymptomatic, the true prevalence may be underestimated, creating a generally hidden burden of disease for the healthcare system (13, 15, 20–22). Therefore, the increasing prevalence of NAFLD is life-threatening, and identifying modifiable risk factors is essential to reduce the disease burden (23). Also, early detection of critical risk factors is the most necessary task now (22). Although the two most common methods to diagnose fatty liver are histological methods and imaging, a diagnostic method has yet to be sufficiently reliable in diagnosing fatty liver (24–26). Although liver biopsy is the gold standard method for the diagnosis of NAFLD, it is also an invasive and expensive tool that has some health risks and economic costs (8, 25–27). Hence, the growing epidemic of NAFLD has led to the need for non-invasive diagnostic measures to predict it (28). Various anthropometric indices can help determine total body fat and fat distribution, including measures focusing specifically on visceral and subcutaneous fat, which is essential to liver health.

Body mass index (BMI) is a primary index for assessing overall obesity. Additionally, waist circumference (WC), mid-arm circumference (MUAC), waist-to-height ratio (WHtR), and waist-to-hip ratio (WHR) are widely recommended for evaluating central body fat distribution. These anthropometric indices provide critical insights into body fat and its distribution patterns, which can be instrumental in predicting the risk of NAFLD (29–32). Therefore, it is possible to prevent NAFLD in its early stages (33). According to reviewed studies (References 29–33), BMI, WC, WHR, and WHtR, preferred body fat indicators, predict NAFLD. Despite all the mentioned cases, so far, a single summary has not been obtained to investigate the relationship between anthropometric indices and NAFLD and introduce a suitable predictive index for this disease. Therefore, we aimed to review the pre-collected data of the FASA Cohort Study (FACS), examining some widely used anthropometric indices related to NAFLD and determining a suitable, reliable, and widely used predictive index for this disease.

## 2 Materials and methods

### 2.1 Study design and population

In the present cross-sectional study, we used the Fasa Cohort Data, which was conducted on about 10,000 people (In this study, NAFLD was confirmed in 1,047 participants through physician evaluations, which relied on medical history and ultrasonographic findings to ensure precise and reliable case identification.), to analyze the predisposing factors of chronic non-communicable diseases common in the rural areas of Fasa City. The Fasa Cohort Study (FACS), which was a population-based study with a 15-year follow-up period, was conducted in a selected rural area known as Sheshda, with a total population of 41,000 people, to evaluate the risk factors associated with predisposing the residents of the rural area of Fasa to diseases. The target population of this study is all men and women aged 35 to 70 (because they were old enough to be exposed to health issues and risk factors and young enough not to have CV and non-communicable diseases) living in the city.

### 2.2 General and anthropometric assessments

First, the general characteristics of the participants were recorded. All registered participants were interviewed to complete a general questionnaire about age, sex, education, job status, physical activity, smoking, alcohol consumption, high blood fat, hypertension, diabetes, history of unwanted weight loss, family history of hypertension, and family history of diabetes. Systolic blood pressure (SBP) and diastolic blood pressure (DBP) were measured using a standard mercury sphygmomanometer in the right arm after 15 min of rest in a sitting position. The first and fifth Korotkoff sounds were considered for SBP and DBP, respectively. The systolic and diastolic blood pressures were measured twice with the mentioned method, and their average was considered. Also, people’s height was measured using a stadiometer with an accuracy of 0.1 cm in a standing position without shoes, heels attached to the wall, and looking forward. In addition, other anthropometric parameters such as weight, total body water content, visceral fat mass, and total body fat mass were measured using bioelectrical impedance analysis (Tanita BC-418, Tanita Corp, Japan) for each participant according to the standard protocol. A flexible tape measure was used to measure WC and hip circumference. WC was measured at a point between the iliac crest and the lowest rib in the standing position, and the hip circumference (HC) was measured at the part of the hip with the maximum circumference. All measurements were read to the nearest 0.1 cm. Then, values of WHR, WHtR, BMI, a body shape index (ABSI), body roundness index (BRI), and visceral fat index (VAI) were also calculated with the help of these measurements and using the following formulas (Equations 1–7):


(1) $$WHR=\frac{WC}{HC}$$

(2) $$\mathrm{WHtR}=\frac{WC}{Ht}$$

(3) $$BMI=\frac{Wt}{Ht^{2}}$$

(4) $$\mathrm{ABSI}=\frac{WC}{\left[BMI^{\frac{2}{3}}\times Ht^{\frac{1}{2}}\right]}$$

(5) $$BRI=364.2-365.5\times {\left(1-\frac{{\left[\frac{WC}{2\pi }\right]}^{2}}{\left[0.5\times Ht^{2}\right]}\right)}^{\frac{1}{2}}$$

(6) $$\mathrm{MEN}:VAI=\left(\frac{WC}{\left(39.68+\left(1.88\times BMI\right)\right)}\right)\times \left(\frac{TG}{1.03}\right)\times \left(\frac{1.31}{HDL}\right)$$

(7) $$\mathrm{WOMEN}:VAI=\left(\frac{WC}{\left(39.58+\left(1.89\times BMI\right)\right)}\right)\times \left(\frac{TG}{0.81}\right)\times \left(\frac{1.52}{HDL}\right)$$

### 2.3 Dietary assessment

The participants completed the Food Frequency Questionnaire (FFQ) to report their dietary habits and the types and amounts of foods they consumed over the past year. Information was entered into the Nutritionist IV package, modified for Iranian foods, to obtain daily energy, nutrient intake, and meals consumed. Food groups and nutrients of mixed foods are calculated based on their ingredients.

### 2.4 Biochemical assessment

Blood samples were taken from all participants to check the following parameters: fasting blood sugar (FBS), serum total cholesterol (Chol), TG, high-density lipoprotein (HDL) and low-density lipoprotein (LDL), alanine aminotransferase (ALT), asparagine aminotransferase (AST), alkaline phosphatase (ALP) and gamma-glutamyl transpeptidase (GGTP).

### 2.5 Statistical analyses

Finally, all the data were analyzed using SPSS software (V.27). Considering that the dependent variable is qualitative, there was no need to check the normality of the data. Data description was done with the help of appropriate descriptive tests (such as t-tests and chi-square tests). These statistical tests were used to compare means between two groups (t-test) and to examine the association between categorical variables (chi-square). Binary logistic regression was used to evaluate the predictive power of each of these indices. This approach was employed to adjust for potential confounding variables (such as age, gender, physical activity, hypertension, diabetes, high blood lipids, energy intake, fat intake, vegetables, and fruit intake), ensuring that extraneous factors did not influence the observed associations between the anthropometric indices and the outcome of interest. By including potential confounders in the model, we aimed to isolate the independent effect of each index on the dependent variable. This statistical adjustment improves the robustness and validity of the results, allowing for more accurate interpretations and minimizing bias due to confounding effects. In addition, ROC curve analysis was also used to comprehensively assess the performance and compare the efficiency of the index, determine their predictive power, and determine the optimal cut-off points for each index (34). Also, the level of *p*-value <0.05 will be considered to show statistical significance (33).

## 3 Results

### 3.1 Study population characteristics

The population of the present cross-sectional study included 10,081 adults aged 35–70, of which 4,539 were men and 5,542 were women. Notably, there are 180 men and 867 women with NAFLD, a significant health concern. The mean (SD) WHR, WHtR, BMI, VAI, ABSI, and BRI in men were 0.91 (0.06), 0.52 (0.06), 24.19 (4.42), 4.39 (3.98), 0.82 (0.04), and 1.34 (0.66), respectively; in women, it is 0.94 (0.06), 0.61 (0.07), 26.86 (4.81), 5.32 (4.20), 0.86 (0.05), and 2.30 (0.88), respectively. The study protocol was approved by the Ethics Committee of Islamic Azad University, Science and Research Unit of Tehran (IR.IAU.SRB.REC.1402.010).

### 3.2 Characteristics of the studied population based on non-alcoholic fatty liver disease among male and female participants

The mean and standard deviation (SD) of quantitative and qualitative variables based on NAFLD among male and female participants are shown in Table 1. The average SBP and DBP in men and women with NAFLD are significantly higher than in their healthy groups (*p* < 0.001). Mean weight, WC, HC, WHR, WHtR, BMI, VAI, and BRI in men and women with NAFLD were significantly higher than in their healthy groups (*p* < 0.001). According to the study, TBF in people with NAFLD is considerably higher than in healthy people (*p* < 0.001). The findings of the survey regarding the lipid profile indicate that the average TG (*p* < 0.001) and TChol (*p* = 0.024 for men and *p* = 0.009 for women) in affected people to NAFLD are significantly higher compared to healthy subjects. In addition, no significant difference was observed in the HDL levels of men with NAFLD and healthy men (*p* = 0.315). However, the difference in HDL levels in women with NAFLD and healthy women was statistically significant (*p* < 0.001). AST and ALT levels in men and women with NAFLD are significantly higher than in their healthy groups (*p* < 0.001).

**Table 1:** Characteristics of the studied population based on non-alcoholic fatty liver disease among male and female participants.

Variable	Men (*N* = 4,539)			Women (*N* = 5,542)		
	With NAFLD	Without NAFLD	*p*-value	With NAFLD	Without NAFLD	*p*-value
	Mean (SD)			Mean (SD)		
**General information**						
Age (Years)	49.06 (8.89)	48.36 (9.31)	0.325	49.75 (8.79)	48.36 (9.52)	**<0.001**
Physical activity (Min per day)	42.5 (13.49)	45.35 (14.36)	0.009	37.35 (5.78)	38.61 (6.88)	**<0.001**
**Anthropometric indices**						
Weight (kg)	79.95 (12.6)	68.8 (13.61)	**<0.001**	72.3 (12.27)	63.98 (12.19)	**<0.001**
Height (cm)	169.05 (5.91)	169.04 (6.5)	0.986	155.89 (5.42)	155.77 (5.75)	0.573
WC (cm)	99.01 (10.15)	89.06 (10.98)	**<0.001**	103.06 (10.51)	94.88 (11.21)	**<0.001**
HC (cm)	102.69 (7.19)	97.34 (7.55)	**<0.001**	105.88 (9.8)	100.41 (9.08)	**<0.001**
WHR (Score)	0.96 (0.05)	0.91 (0.06)	**<0.001**	0.97 (0.05)	0.94 (0.06)	**<0.001**
WHtR (Score)	0.58 (0.06)	0.52 (0.06)	**<0.001**	0.66 (0.06)	0.6 (0.07)	**<0.001**
BMI (kg/m2)	27.95 (4.09)	24.04 (4.36)	**<0.001**	29.73 (4.69)	26.33 (4.64)	**<0.001**
VAI (Score)	5.89 (5.26)	4.33 (3.91)	**<0.001**	6.55 (5.16)	5.09 (3.96)	**<0.001**
ABSI (Score)	0.82 (0.03)	0.82 (0.04)	0.32	0.86 (0.04)	0.86 (0.05)	0.617
BRI (Score)	1.93 (0.7)	1.32 (0.65)	**<0.001**	2.82 (0.85)	2.21 (0.85)	**<0.001**
**Blood pressure**						
SBP (mmHg)	117.01 (16.71)	110.09 (17.28)	**<0.001**	114.58 (18.43)	111.59 (19.19)	**<0.001**
DBP (mmHg)	80.07 (12.72)	74.07 (11.65)	**<0.001**	76.84 (11.83)	74.53 (12.04)	**<0.001**
Pulse rate (beats per min)	73.61 (10.22)	71.18 (10.13)	0.002	76.3 (10.75)	76.35 (10.52)	0.89
**Body composition**						
TBF (kg)	21.22 (7)	14.04 (7.27)	**<0.001**	28.05 (7.77)	22.09 (8.1)	**<0.001**
TBW (Lit)	43.83 (5.32)	40.001 (5.57)	**<0.001**	32.63 (3.31)	30.88 (3.52)	**<0.001**
**Blood parameters**						
FBS (mg/dl)	102 (40.95)	89.67 (23.19)	**<0.001**	100.1 (37.9)	93.54 (31.95)	**<0.001**
TG (mg/dl)	173.27 (131.4)	134.91 (89.3)	**<0.001**	151.47 (90.98)	124.001 (69.78)	**<0.001**
TChol (mg/dl)	187.51 (53.13)	178.39 (37.12)	**0.024**	193.47 (41.57)	189.68 (38.93)	**0.009**
HDL (mg/dl)	46 (16.61)	47.08 (14.14)	0.315	52.06 (15.66)	54.46 (16.43)	**<0.001**
LDL (mg/dl)	106.86 (38.34)	104.42 (31.13)	0.402	111.43 (35.02)	110.54 (32.88)	0.489
AST (IU/lit)	26.07 (9.23)	23.76 (8.35)	**0.001**	22.62 (10.19)	21.31 (8.02)	**<0.001**
ALT (IU/lit)	34.79 (19.22)	25.79 (16.29)	**<0.001**	24.61 (13.06)	20.69 (11.66)	**<0.001**
ALP (IU/lit)	227.88 (152.69)	210.04 (59.01)	0.12	219.29 (98.5)	205.84 (70.07)	**<0.001**
GGTP (IU/lit)	35.88 (44.91)	25.36 (20.95)	**0.002**	25.03 (28.35)	19.58 (17.94)	**<0.001**
Qualitative variables						
**Unwanted weight loss**						
Yes	7 (3.9%)	98 (2.2%)	0.151	15 (1.7%)	72 (1.5%)	0.679
No	173 (96.1%)	4,261 (97.8%)		852 (98.3%)	4,603 (98.5%)	
**Education**						
Illiterate	60 (33.3%)	1,487 (34.1%)	**<0.001**	485 (55.9%)	2,563 (54.8%)	0.467
Elementary school	49 (27.2%)	1,414 (32.4%)		283 (32.6%)	1,545 (33%)	
Middle school	30 (16.7%)	889 (20.4%)		68 (7.8%)	371 (7.9%)	
High school diploma	27 (15%)	418 (9.6%)		20 (2.3%)	138(3%)	
Associate degree	5 (2.8%)	42 (1%)		1 (0.1%)	13 (0.3%)	
Bachelor’s degree	5 (2.8%)	93 (2.1%)		8 (0.9%)	43 (0.9%)	
Master’s degree	3 (1.7%)	16 (0.4%)		2 (0.2%)	2 (0%)	
Doctorate (PhD)	1 (0.6%)	0 (0%)		0 (0%)	0 (0%)	
**Job**						
Employed	145 (80.6%)	3,790 (87.1%)	**0.011**	130 (15%)	1,022 (21.9%)	**<0.001**
Unemployed	35 (19.4%)	563 (12.9%)		736 (85%)	3,642 (78.1%)	
**Smoking**						
Yes	76 (42.2%)	2,382 (54.6%)	**0.001**	50 (5.8%)	226 (4.8%)	0.246
No	104 (57.8%)	1977 (45.4%)		817 (94.2%)	4,449 (95.2%)	
**Alcohol consumption**						
Yes	14 (7.8%)	470 (10.8%)	0.201	0 (0%)	1 (0%)	0.667
No	166 (92.2%)	3,889 (89.2%)		867 (100%)	4,674 (100%)	
Family history						
**Hypertension**						
Yes	41 (22.8%)	464 (10.6%)	**<0.001**	362 (41.8%)	1,135 (24.3%)	**<0.001**
No	139 (77.2%)	3,895 (89.4%)		505 (58.2%)	3,540 (75.7%)	
**Diabetes**						
Yes	46 (25.6%)	306 (7%)	**<0.001**	248 (28.6%)	645 (13.8%)	**<0.001**
No	134 (74.4%)	4,053 (93%)		619 (71.4%)	4,030 (86.2%)	

NAFLD, Non-alcoholic fatty liver disease; WC, Waist circumference; HC, Hip circumference; WHR, Waist circumference to hip circumference ratio; WHtR, Waist circumference to height ratio; BMI, Body mass index; VAI, Visceral adiposity index; ABSI, A body shape index; BRI, Body roundness index; SBP, Systolic blood pressure; DBP, Diastolic blood pressure; TBF, Total body fat; TBW, Total body water; FBS, Fasting blood sugar; TG, Triglycerides; TChol, Total serum cholesterol; HDL, High density lipoprotein; LDL, Low density lipoprotein; AST, Asparagine aminotransferase; ALT, Alanine aminotransferase; ALP, Alkaline phosphatase; GGTP, Gammaglutamyl transpeptidase, with NAFLD, Men = 180 and women = 867, Without NAFLD, Men = 4,359 and women = 4675. *p*- value < 0.05 is considered significant. Bold values represent statistically significant results.

Also, data related to smoking shows that there are significant differences between affected and healthy groups in men (*p* < 0.001), but this difference is not observed in women (*p* = 0.246). Analysis of family history data shows significant differences in family history of blood pressure and diabetes between affected and healthy groups in both sexes (*p* < 0.001).

### 3.3 Dietary intakes based on non-alcoholic fatty liver disease

The mean (SD) of energy and dietary intake of the participants based on non-alcoholic fatty liver disease among male and female participants are shown in Table 2. Notably, in men, the average daily intake of vegetables in patients with NAFLD compared to their healthy group was statistically significant (*p* = 0.015), indicating a potential dietary factor in NAFLD. In women, several nutritional factors were significantly lower in patients with NAFLD than in their healthy group, including total fat (*p* = 0.003), saturated fat (*p* = 0.005), MUFA (*p* = 0.029), and fruits (*p* < 0.001).

**Table 2:** Dietary intakes of the studied population based on non-alcoholic fatty liver disease among male and female participants.

Variable	Men (*n* = 4,539)			Women (*n* = 5,542)		
	With NAFLD	Without NAFLD	*p*- value	With NAFLD	Without NAFLD	*p*- value
	Mean (SD)			Mean (SD)		
Energy (kcal/day)	3040.74 (1244.45)	3053.27 (1169.9)	0.888	2806.38 (1118.35)	2850.22 (1112.33)	0.288
Total fat (gr/day)	73.09 (31.03)	73.08 (29.98)	0.997	65.96 (28.33)	69.2 (29.65)	**0.003**
Trans fat (gr/day)	0.4 (0.56)	0.34 (0.37)	0.125	0.29 (0.36)	0.31 (0.37)	0.331
Saturated fatty acid (gr/day)	25.14 (12.13)	25.5 (11.97)	0.691	23.19 (11.97)	24.45 (12.08)	**0.005**
MUFA (gr/day)	19.59 (9.56)	19.32 (9.12)	0.7	17.54 (8.45)	18.26 (9.002)	**0.029**
PUFA (gr/day)	11.22 (5.65)	10.62 (5.52)	0.159	9.79 (4.87)	9.95 (5.14)	0.417
Vegetables (gr/day)	667.89 (398.02)	593.66 (337.97)	**0.015**	587.75 (316.24)	573.55 (318.27)	0.228
Fruits (gr/day)	483.75 (355.58)	433.98 (359.55)	0.069	413.4 (325.1)	355.96 (280.04)	**<0.001**

NAFLD, Non-alcoholic fatty liver disease; MUFA, Mono unsaturated fatty acids, PUFA: Polyunsaturated fatty acids. With NAFLD: Men = 180 and women = 867, Without NAFLD: Men = 4,359 and women = 4675. *p*- value < 0.05 is considered significant. Bold values represent statistically significant results.

### 3.4 Association between NAFLD and WHR, WHtR, BMI, VAI, ABSI, BRI

In both crude and adjusted models (adjusted for confounding factors such as age, gender, physical activity, hypertension, diabetes, high blood lipids, energy intake, fat intake, vegetables, and fruit intake), binary logistic regression was used to investigate the association between anthropometric indices and the odds of NAFLD (Table 3).

**Table 3:** Predictive Accuracy of WHR, WHtR, BMI, VAI, ABSI, and BRI for NAFLD in Crude and Adjusted models.

Variables	With NAFLD (*N* = 1,047)							
	Crude model				Adjusted model			
	*β*	OR	95% CI	*p*- value	*β*	OR	95% CI	*p*- value
WHR	1.07	2.94	2.64–3.27	**<0.001**	0.3	1.35	1.12–1.64	**0.002**
WHtR	1.15	3.16	2.9–3.43	**<0.001**	2.79	16.3	4.27–62.24	**<0.001**
BMI	0.17	1.18	1.17–1.20	**<0.001**	0.13	1.14	1.06–1.24	**<0.001**
VAI	0.06	1.07	1.05–1.08	**<0.001**	0.03	1.03	1.01–1.04	**<0.001**
ABSI	4.62	101.73	30.34–341.04	**<0.001**	0.65	1.93	0.02–150.05	0.767
BRI	0.93	2.55	2.38–2.73	**<0.001**	−2.35	0.09	0.03–0.25	**<0.001**

WHR, Waist-to-Hip Ratio; WHtR, Waist-to-Hight Ratio; BMI, Body Mass Index; VAI, Visceral Adiposity Index; ABSI, A Body Shape Index; BRI, Body Roundness Index; OR, Odds Ratio; CI, Confidence Interval. All data are presented as OR and 95% CI by binary logistic regression. Adjusted model: based on age, gender, physical activity, hypertension, diabetes, hyperlipidemia, energy intake, fat intake, vegetables and fruit intake. People who did not suffer from NAFLD were considered as the reference group. *p*- value < 0.05 is considered significant. Bold values represent statistically significant results.

In the crude and adjusted model, there was a positive and significant relationship between WHR (crude model: OR = 2.94, 95% CI = 2.64–3.27, *p* < 0.001; adjusted model: OR = 0.3, 95% CI = 1.12–1.64, *p* = 0.002), WHtR (crude model: OR = 3.16, 95% CI = 2.9–3.43, *p* < 0.001; adjusted model: OR = 16.3, 95% CI = 4.27–62.24, *p* < 0.001), BMI (crude model: OR = 1.18, 95% CI = 1.17–1.20, *p* < 0.001; adjusted model: OR = 1.14, 95% CI = 1.06–1.24, *p* < 0.001) and VAI (crude model: OR = 1.07, 95% CI = 1.05–1.08, *p* < 0.001; adjusted model: OR = 1.03, 95% CI = 1.01–1.04, *p* < 0.001) and odds of NAFLD. In the crude model, the odds ratio (OR) for ABSI was significant (OR = 101.73, 95% CI = 30.34–341.04, *p* < 0.001), indicating a strong positive and significant relationship between ABSI and the odds of NAFLD. However, in the adjusted model, OR decreased to 1.93, and the *p*-value was out of the significant level (95% CI = 0.02–150.05, *p* = 0.767). In the crude model, BRI was positively and significantly associated with the odds of NAFLD (OR = 2.55, 95% CI = 2.38–2.73, *p* < 0.001); this means that with the increase of each unit of BRI, the probability of NAFLD increases by 2.55 times. However, after adjustment, the relationship between BRI and the odds of NAFLD changed significantly, and the OR decreased to 0.09, and *β* became negative (95%CI = 0.03–0.25, *p* < 0.001).

### 3.5 Comparison of sensitivity and specificity of WHR, WHtR, BMI, VAI, ABSI and BRI in predicting NAFLD in men and women using ROC curve analysis

**Figure 1:**
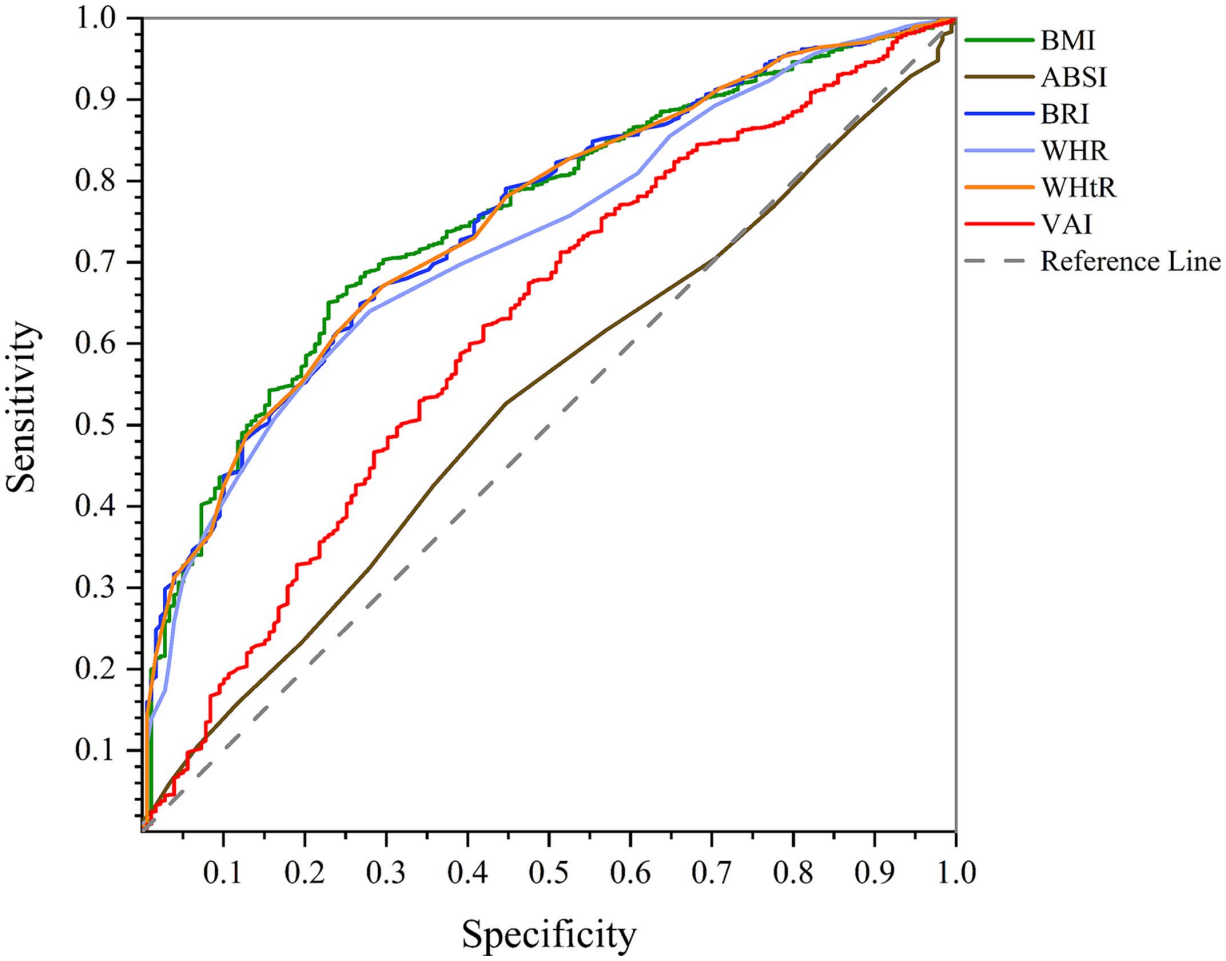
Comparison of anthropometric indices in terms of predicting NAFLD in men using AUC of ROC curve.

**Figure 2:**
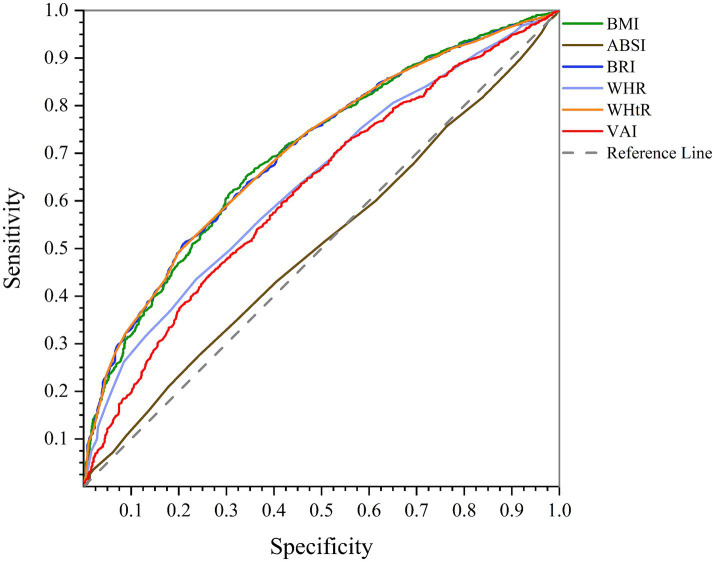
Comparison of anthropometric indices in terms of predicting NAFLD in women using AUC of ROC curve.

According to AUC of ROC curve analysis, the results of this study clearly show that WHtR (men: AUC = 0.750, 95%CI = 0.717–0.782, *p* < 0.001; women: AUC = 0.702, 95%CI = 0.684–0.720, *p* < 0.001) and BMI (men: AUC = 0.754, 95%CI = 0.722–0.786, *p* < 0.001; women: AUC = 0.701, 95%CI = 0.683–0.719, *p* < 0.001) are the best predictors of NAFLD in both genders. While BRI (men: AUC = 0.750, 95%CI = 0.717–0.782, *p* < 0.001; women: AUC = 0.702, 95%CI = 0.684–0.720, *p* < 0.001) also has significant accuracy, WHR (men: AUC = 0.727, 95%CI = 0.694–0.760, *p* < 0.001; women: AUC = 0.640, 95%CI = 0.621–0.659, *p* < 0.001) and VAI (men: AUC = 0.621, 95%CI = 0.578–0.663, *p* < 0.001; women: AUC = 0.622, 95%CI = 0.602–0.640, *p* < 0.001) are next. On the other hand, ABSI (men: AUC = 0.530, 95%CI = 0.489–0.570, *p* = 0.178; women: AUC = 0.505, 95%CI = 0.485–0.526, *p* = 0.611) is known to be a poor predictor of NAFLD and is not recommended. These findings can help design more effective strategies for NAFLD screening and management (Figures 1, 2; Table 4).

**Table 4:** Comparison of sensitivity and specificity of WHR, WHtR, BMI, VAI, ABSI and BRI in predicting NAFLD in men and women using ROC curve analysis.

Variables	With NAFLD					
	Men (*N* = 180)			Women (*N* = 867)		
	AUC (SE)	95% CI	*p*- value	AUC (SE)	95% CI	*p*- value
WHR	0.727 (0.01)	0.694–0.76	**<0.001**	0.64 (0.01)	0.621–0.659	**<0.001**
WHtR	0.75 (0.01)	0.717–0.782	**<0.001**	0.702 (0.009)	0.684–0.72	**<0.001**
BMI	0.754 (0.01)	0.722–0.786	**<0.001**	0.701 (0.009)	0.683–0.719	**<0.001**
VAI	0.621 (0.01)	0.578–0.663	**<0.001**	0.622 (0.01)	0.602–0.64	**<0.001**
ABSI	0.53 (0.02)	0.489–0.57	0.178	0.505 (0.01)	0.485–0.526	0.611
BRI	0.75 (0.01)	0.717–0.782	**<0.001**	0.702 (0.009)	0.684–0.72	**<0.001**

WHR, Waist-to-Hip Ratio; WHtR, Waist-to-Hight Ratio; BMI, Body Mass Index; VAI, Visceral Adiposity Index; ABSI, A Body Shape Index; BRI, Body Roundness Index. AUC, Area under Curve; CI, Confidence Interval. *p*-value < 0.05 is considered significant. Bold values represent statistically significant results.

### 3.6 Determining the cut-points of anthropometric indices in men and women using the YOUDEN index

The cut-points determined based on the Youden index represent the optimal values for separating people with NAFLD from healthy people. Among the examined indices, BMI (cut-points = men: 25.55, women: 27.74), with the highest Youden index in both sexes, is the most accurate predictive tool, while ABSI (cut-points = men: 0.82, women: 0.83) is the least accurate. A cut-point of 25.55 for BMI indicates an increased risk of developing NAFLD in men whose BMI is more significant than this value. This cut point balances sensitivity (77%) and specificity (34%). In women, a cut-point for a BMI of 27.74 with a Youden index of 1.311 provides the best balance between sensitivity and specificity, indicating that women with a BMI greater than 27.74 are more likely to develop NAFLD. ABSI higher than 0.82 is associated with an increased probability of NAFLD in men. Still, low sensitivity (55%) and a relatively low Youden index (1.080) indicate that it needs to be more accurate than other indices. On the other hand, in ABSI women with a cut-off point of 0.83, although it has relatively high sensitivity and specificity (75 and 72%), the Youden index of 1.032 shows that this index is less accurate in predicting NAFLD compared to other indices (Table 5).

**Table 5:** Determining the cut-points of anthropometric indices in men and women using the YOUDEN index.

Variables		Cut points	Sensitivity	Specificity	Max YOUDEN index
WHR	Men	0.93	0.73	0.36	1.369
	Women	0.93	0.78	0.58	1.202
WHtR	Men	0.55	0.73	0.34	1.384
	Women	0.6	0.79	0.49	1.301
BMI	Men	25.55	0.77	0.34	1.422
	Women	27.74	0.66	0.34	1.311
		27.77	0.65	0.34	
VAI	Men	4.05	0.34	0.37	1.204
	Women	3.75	0.73	0.55	1.181
ABSI	Men	0.82	0.55	0.47	1.080
	Women	0.83	0.75	0.72	1.032
BRI	Men	1.52	0.73	0.35	1.381
	Women	2.14	0.79	0.49	1.3

WHR, Waist-to-Hip Ratio; WHtR, Waist-to-Hight Ratio; BMI, Body Mass Index; VAI, Visceral Adiposity Index; ABSI, A Body Shape Index; BRI, Body Roundness Index. The color shade presented is green.

## 4 Discussion

Several studies show that the global prevalence of NAFLD is about 25 to 30%. According to one comprehensive review, the global prevalence of NAFLD in adults has been estimated at 32%, which is higher in men (40%) than women (26%) (35). A review and meta-analysis study in Iran showed that the prevalence of NAFLD in the general population of Iran is about 33.9%, which is higher than in some other countries (36). In our study population, the prevalence of NAFLD in adults aged 35–70 is about 10.3%, of which about 82.3% are women and the rest are men.

The study’s results clearly show that patients with NAFLD have different and significant physical and biochemical characteristics compared to healthy groups. Specifically, older age, less physical activity, hypertension, and a significant increase in pulse rate have been observed in these patients. In addition, the values of weight, obesity indices, and TBF in people with NAFLD are significantly higher than in healthy groups, and the amount of TBW is also reduced in these people. FBS, TG, and TChol are increased dramatically in patients with NAFLD, and the levels of liver enzymes, including AST, ALT, and GGTP, are also significantly higher in these groups than in healthy groups. In addition, significant differences were observed in the level of education, job status, and smoking between people with NAFLD and healthy groups. Also, the analysis of family records of hypertension and diabetes showed a significant difference in the incidence of NAFLD between these two groups. In addition, the results of dietary intakes showed that in men with NAFLD, the only significant mean difference is related to the daily intake of vegetables, which is less than the healthy group.

On the contrary, the significant mean difference in women with NAFLD is associated with the daily intake of total fat, saturated fat, MUFA, and fruits, which is lower than healthy groups. The Food Frequency Questionnaire (FFQ) can be influenced by recall bias, as participants may underreport or overreport their intake, leading to inaccuracies in the collected data. Additionally, FFQs may lack sensitivity to variations in portion sizes, further decreasing the accuracy of dietary assessments. Moreover, dietary homogeneity within the sample group and socioeconomic or cultural constraints might limit observed differences in dietary intake. Although statistically significant differences were found in the study, these factors may help explain why clinically relevant differences were not apparent.

Moreover, this study employed binary logistic regression to investigate various anthropometric indices, including BMI, WHR, WHtR, VAI, ABSI, and BRI, as the leading independent variables. Binary logistic regression allowed us to effectively investigate the relationship between these indices and NAFLD, providing important findings contributing to our understanding of this health issue.

The results of the current cross-sectional study showed that in both crude and adjusted models, there was a positive and significant relationship between WHR, WHtR, BMI, VAI, and BRI with the odds of NAFLD. However, results were different for the ABSI; in the crude model, OR was significantly high and showed a strong relationship with the odds of NAFLD, while in the adjusted model, this relationship decreased and was out of the statistical significance level. Fujita et al.’s study confirmed our findings and showed that ABSI served as a strong predictor of diabetes, hypertension, and dyslipidemia. After adjusting for confounding variables, ABSI’s predictive power decreased and, in some cases, became statistically insignificant. This shows a significant influence of moderating variables on the relationship between ABSI and health outcomes (37).

These indices usually indicate obesity and fat accumulation in the abdominal area. Visceral fat or abdominal fat has been directly associated with inflammation, insulin resistance, increased risk of type 2 diabetes, and oxidative stress, which leads to fat accumulation in the liver, destruction of liver cells, and increased risk of NAFLD (38–40). Indices such as BMI and WHtR are associated with an increased risk of cardiovascular diseases, which can be related to an increased risk of NAFLD, as hypertension and increased lipids are aggravating factors of this disease (41). Studies have shown that increased WC, WHtR, WHR, BMI, and VAI are significantly associated with increased risk of NAFLD in both men and women. These indices are significant in people with visceral obesity (fat accumulation in the abdominal area). They are critical indices in predicting NAFLD because of their direct effect on fat accumulation in the liver (40, 42). A study showed that combining anthropometric indices, such as BMI, WHR, or VAI, could better predict the risk of developing NAFLD. In these studies, WHtR has been identified as a simple but effective tool to identify individuals at high risk of NAFLD (38). Another survey of the association between central obesity and NAFLD showed that central obesity, measured by WC and WHtR, was strongly associated with NAFLD. This study also showed that WHtR is one of the best predictors of NAFLD (42). Analysis of risk factors in type 2 diabetic patients in a study showed that patients with type 2 diabetes with high BMI, WC, and WHR are more at risk of developing NAFLD.

Furthermore, increased WHR and VAI were identified as substantial risk factors for NAFLD in these patients (39). One study investigated metabolic risk factors and their association with NAFLD progression. The results showed that obesity indices, such as BMI and WHtR, are associated with an increased risk of NAFLD and its progression to more severe diseases, such as cirrhosis and liver cancer (43). A study of more than 14,000 participants found that ABSI was a poor predictive index for NAFLD and was not significantly associated with NAFLD compared to other indices, such as BMI, WHtR, and VAI (44). The study of Vongsuvanh et al. showed that ABSI was not significantly related to the severity of steatosis, inflammation, or liver fibrosis in patients with NAFLD. In this study, ABSI did not perform considerably in predicting NAFLD, and other indices, such as WC and BMI, performed better (45). In the study of Bouchi et al., the relationship of ABSI with arterial stiffness and other cardiovascular parameters in type 2 diabetic patients was investigated. The results showed that ABSI has no significant relationship with many of these profiles, including NAFLD, while other profiles, such as WC and BMI, showed a better relationship (46). Comparing the findings of our study with those of systematic reviews shows that changes in body composition, such as a reduction in visceral fat, are significantly associated with improvements in liver steatosis in NAFLD. A meta-analysis has shown that changes in indices such as BMI and WHtR can reduce disease progression and substantially improve liver status (47). These findings are consistent with our study results regarding the role of these indices in predicting the risk of NAFLD and its progression (48). A study by Mátis et al. (16) systematically reviewed the impact of body composition changes on liver fat content in patients with NAFLD. This review revealed a moderate correlation between changes in visceral adipose tissue (VAT) and improvements in liver steatosis. Specifically, a pooled correlation coefficient of r = 0.49 (CI: 0.22–0.69) was observed between VAT change and steatosis improvement based on two studies with 85 patients. This suggests that targeting visceral fat reduction could potentially aid in the resolution of liver fat accumulation in NAFLD (16).

Various studies have shown that lifestyle interventions, such as dietary changes and regular physical activity, can significantly improve body composition and reduce liver steatosis. For example, digital programs supporting lifestyle changes, including reduced calorie intake and increased physical activity, have led to a decrease in BMI and improved liver enzymes in NAFLD patients (49). These results align with nutritional interventions, such as the Mediterranean diet and low-fat diets, which play a role in improving the metabolic status of NAFLD patients (50). Additionally, interventions based on digital technologies and support from lifestyle coaches can be valuable tools for sustainably implementing these changes (51). Huang et al. conducted a study in which participants received intensive nutritional counseling over 1 year, aiming to improve histological outcomes in patients with NASH (Non-Alcoholic Steatohepatitis). They found significant improvements in liver function, particularly in reducing the NASH score, indicating the potential benefits of dietary adjustments in managing liver fat content and improving liver health (52).

This study used the ROC curve to investigate the effectiveness of six anthropometric indices (WHR, WHtR, BMI, VAI, ABSI, and BRI) in predicting NAFLD in men and women. According to the ROC curve analysis results, BMI is the strongest predictor of NAFLD in men, and WHtR and BRI are the strongest predictors of NAFLD in women. In addition, according to the results, ABSI has the weakest performance in predicting NAFLD, both in men and women. Past studies confirm that WHtR and BMI are key indices in predicting NAFLD. BRI is also known as a valid index, and ABSI performs weaker in predicting this disease than other indexes (53–55).

In this study, cut points were determined using the Youden index to evaluate the performance of different anthropometric indices. This method is beneficial when deciding which cut-point results in the best combination of sensitivity and specificity. By calculating the Youden index for each cut point, the point with the highest index value is selected as the optimal cut point. This cut point offers the best balance between sensitivity and specificity. We made sensitivity-based decisions where multiple cut points had the same Youden index value. We chose the cut-off point with the highest sensitivity to ensure maximum identification of positive cases, which is particularly useful in situations where accurate disease diagnosis is essential (56, 57). The investigations carried out on previous studies that calculated the cut points of these indices using the Youden index indicate that the cut points of the indices are different depending on the population and conditions. Each index can help predict certain risks such as MetS, CVDs, NAFLD, and other diseases. To a large extent, the cut points reported in previous studies confirm the results related to calculating the cut points of anthropometric indices in our study. The slight difference in the values of the cut points is due to the difference in the population and disease studied (29, 58–66).

Future studies could focus on changes in anthropometric indices over time and examine the impact of these changes on progression or improvement. These investigations can help in timely preventive interventions. Also, the study of gender and ethnic differences in NAFLD can show that men, women, or different ethnic groups respond differently to risk factors, which will help improve health and treatment policies. In addition, future studies should examine the effects of therapeutic interventions and lifestyle changes, such as weight loss and specific diets, on anthropometric indices and NAFLD progression to provide more information on the impact of these changes on disease improvement.

### 4.1 Strengths and limitations

With access to high-quality data and a large sample size (about 10,000 people), this study has enabled robust statistical analysis and valid results. Ethnic diversity (Persian, Turkish, and Arab) helps to generalize findings and reduce genetic and cultural biases. A comprehensive review of various anthropometric indices allows the identification of the best indices for predicting NAFLD. Determining optimal cut-off points using ROC analysis and evaluation of moderating factors increases the accuracy and applicability of the results. These findings can be used in the design of NAFLD screening and management programs. Due to its cross-sectional nature, this study cannot prove the causal relationship between anthropometric indices and NAFLD, and only relationships between variables can be investigated. In addition, although this research is population-based, its focus on a specific rural area in southern Iran limits the generalization of the results to other populations, such as urban populations or different geographic regions. One of the main challenges of the study is the use of the data of an existing cohort, which may need to be revised in the analysis of the results due to the impossibility of complete control over the way of data collection and the selection of variables. Also, in long-term cohort studies, there is a possibility of losing participants due to reasons such as death, migration, or unwillingness to continue cooperation, which can reduce the statistical power and generalizability of the results. Finally, some information was collected through self-reporting, which could lead to reporting bias or errors. Also, FFQ can be influenced by recall bias, as participants may underreport or over report their intake, leading to inaccuracies in the collected data. Additionally, FFQs may lack sensitivity to variations in portion sizes, further decreasing the accuracy of dietary assessments. Moreover, dietary homogeneity within the sample group and socioeconomic or cultural constraints might limit observed differences in dietary intake.

## 5 Conclusion

This research shows that anthropometric indices have a significant relationship with NAFLD. Among the investigated indicators, BMI in men and BRI and WHtR in women have the most potential to predict NAFLD. In both genders, ABSI is the least effective in identifying NAFLD. Optimal cut-points were also calculated for each anthropometric index (WHR, WHtR, BMI, VAI, ABSI, and BRI) in men and women separately. These findings emphasize the importance of using anthropometric indices in identifying and managing NAFLD and can help improve prevention and treatment strategies.

## Data Availability

The data analyzed in this study is subject to the following licenses/restrictions: Data sharing not applicable to this article as no datasets were generated or analyzed during the current study. The general information is available from: http://ncdrc.fums.ac.ir/. Requests to access these datasets should be directed to: http://ncdrc.fums.ac.ir/.
